# Measuring physical activity-related environmental factors: reliability and predictive validity of the European environmental questionnaire ALPHA

**DOI:** 10.1186/1479-5868-7-48

**Published:** 2010-05-26

**Authors:** Heleen Spittaels, Maïté Verloigne, Christopher Gidlow, Julien Gloanec, Sylvia Titze, Charlie Foster, Jean-Michel Oppert, Harry Rutter, Pekka Oja, Michael Sjöström, Ilse De Bourdeaudhuij

**Affiliations:** 1 Department of Movement and Sports Sciences, Ghent University, Watersportlaan 2, 9000 Ghent, Belgium; 2 Centre for Sport and Exercise Research, Staffordshire University, Leek Road, Stoke-on-Trent, Staffordshire ST4 2DF, UK; 3 Nutritional Epidemiology Unit, UMR INSERM U557/INRA U1125/CNAM/University Paris 13, CRNH IdF, 93017 Bobigny, France; 4 Department of Nutrition, Pitie-Salpetriere Hospital (AP-HP), University Pierre et Marie Curie-Paris6, CRNH IdF, 75013 Paris, France; 5 Institute of Sports Science, University of Graz, Mozartgasse 14, 8010 Graz, Austria; 6 Department of Public Health, University of Oxford, Old Road Campus, Headington, Oxford OX3 7LF, UK; 7 National Obesity Observatory, 4150 Chancellor Court, Oxford OX4 2GX, UK; 8 Urho Kaleva Kekkonen Institute for Health Promotion Research, FIN-33500 Tampere, Finland; 9 Karolinska Institute, Department of Biosciences, Preventive Nutrition, Novum, 141 57 Huddinge, Sweden

## Abstract

**Background:**

A questionnaire to assess physical activity related environmental factors in the European population (a 49-item and an 11-item version) was created as part of the framework of the EU-funded project "Instruments for Assessing Levels of PHysical Activity and fitness (ALPHA)". This paper reports on the development and assessment of the questionnaire's test-retest stability, predictive validity, and applicability to European adults.

**Methods:**

The first pilot test was conducted in Belgium, France and the UK. In total 190 adults completed both forms of the ALPHA questionnaire twice with a one-week interval. Physical activity was concurrently measured (i) by administration of the long version of the International Physical Activity Questionnaire (IPAQ) by interview and (ii) by accelerometry (Actigraph™ device). After adaptations, the second field test took place in Belgium, the UK and Austria; 166 adults completed the adapted questionnaire at two time points, with minimum one-week interval. In both field studies intraclass correlation coefficients (ICC) and proportion of agreement were computed to assess the stability of the two test scores. Predictive validity was examined in the first field test by correlating the results of the questionnaires with physical activity data from accelerometry and long IPAQ-last 7 days.

**Results:**

The reliability scores of the ALPHA questionnaire were moderate-to good in the first field testing (ICC range 0.66 - 0.86) and good in the second field testing (ICC range 0.71 - 0.87). The proportion of agreement for the ALPHA short increased significantly from the first (range 50 - 83%) to the second field testing (range 85 - 95%). Environmental scales from both versions of the ALPHA questionnaire were significantly associated with self-reported minutes of transport-related walking, and objectively measured low intensity physical activity levels, particularly in women. Both versions were easily administered with an average completion time of six minutes for the 49-item version and less than two minutes for the short version.

**Conclusion:**

The ALPHA questionnaire is an instrument to measure environmental perceptions in relation to physical activity. It appears to have good reliability and predictive validity. The questionnaire is now available to other researchers to investigate its usefulness and applicability across Europe.

## Background

Until recently, physical activity promotion research has focused on individual factors (demographics and psychosocial determinants). There is now growing agreement among researchers that the physical or built environment may play an important role as well [1,2]. Research into the link between the built environment and physical activity is still in its infancy, but is expanding rapidly as demonstrated by the Active Living Research Reference list that comprised 465 references, published in 2008 in various journals [3,4].

However, until now, evidence of the predictive relationship between environmental determinants and physical activity is not very consistent. Wendel-Vos and colleagues found in their review of 47 papers [5] only a few consistent correlates among adults, e.g. between availability of physical activity equipment and vigorous physical activity and between trailconnectivity and active commuting. Also in youth, only some specific consistent associations were found between environmental factors and physical activity [6].

One of the challenges of this new research domain is how to measure attributes of the built environment associated with physical activity in a valid, reliable and feasible way. Studies of the physical environment and physical activity have typically used two types of exposure measures: (i) measures of perceptions of the environment using questionnaires; (ii) objective measures of the environment derived from observations of the environment (audits, ground truthing) or through spatial Geographic Information Systems (GIS) data [7].

Early measures of perceptions of the environment were criticised for their lack of metric data (e.g. repeatability, face validity) [8]. The development of perceived environmental measures has emerged outside Europe: in Australia the Social Environmental Individual Determinants (SEID) study conducted by Giles-Corti and colleagues [9] and from three US research centres in North Carolina [10], South Carolina [11], and California [12]. Characteristics of the built environment in Europe differ considerably from those in the US or Australia, especially in terms of housing density and land use mix. This raises questions about the applicability of these questionnaires in a European context. As a consequence a small number of European studies have developed their own or have adapted international questionnaires to the European context. However, a consensus about which environmental questionnaire should be used in Europe has yet to be reached.

One objective of the EU-funded Instruments for Assessing Levels of Physical Activity and Fitness (ALPHA) project, is to propose standardised instruments for physical activity and fitness monitoring across Europe [13]. On the basis of a literature review on currently used environmental questionnaires in Europe and a consensus meeting with an international expert group, a European environmental questionnaire was conceived [14]. Two versions were developed: a form containing 49 items suitable for use in research studies and a shorter 11-item form more suitable for surveillance and monitoring purposes. The development of the questionnaire is described in more detail elsewhere [14]. The next step in the project was to test the reliability and validity of the questionnaire in different languages and in different European countries. The paper reports on assessment of the test-retest stability, predictive validity and feasibility of the ALPHA environmental questionnaire in three European countries.

## Methods

The reliability and validity testing were undertaken in four phases, translation, cognitive testing, and two iterations of field testing. First of all the original version of the ALPHA questionnaire was translated into Dutch, French, and German, followed by cognitive testing. Next a first field test was conducted in three countries. An expert meeting was organised to discuss the results before a second smaller field test was conducted to assess the modified questionnaire.

### Translation and cognitive testing

The English questionnaire (the source) was translated into Dutch, French and German using a standard protocol based on the guidelines of Eurostat [15]. To guide the translation process, conceptual cards were included after each question in the English version. These conceptual cards contained brief notes to explain the format of the questions and the underlying concept to be measured. Two translators, both of whom were native speakers and familiar with the topic, worked independently. They read and translated these conceptual cards into the target language before translation of the questions. After translation the two translators, together with a reviewer, discussed any particular translation problems until a final consensus was reached.

After the translation process, cognitive testing was conducted using cognitive interviewing [16] with at least five persons for each language. Respondents were asked to think aloud while processing each question and deciding how to answer to the question. If something was not clear the interviewer would ask questions to start a discussion.

Through the cognitive testing process, questions that were not clear or comprehensive were identified, discussed with the research team and rephrased.

### Field testing I

#### Participants and procedures

Participants were recruited in three countries (Belgium, UK and France) between October 2008 and January 2009. To ensure some variance in the measured characteristics (e.g. population density), the participants within each country were derived from distinct areas (and thus different built environments). In Belgium a random sample in three different neighbourhoods (town, outskirts of town and village/countryside) was drawn. In each neighbourhood, letters with information about the study were distributed by post. One week after mailing the information letter, potential participants were visited at home and asked if they would participate. In the UK, participants randomly selected from 10 areas of an English city for a previous study [17], were contacted by telephone and appointments arranged to visit willing individuals. In France a convenience sample of adults living in the city centre and suburbs of Paris was recruited. Inclusion criteria were: aged 20-65 years, literate in the language of the questionnaire (Dutch, English or French respectively), lived at their current address for at least two months, and without physical disability that would prevent or hamper walking or cycling. The final sample consisted of 190 participants, 60 from Belgium, 64 from UK and 66 from France.

To assess test-retest stability, participants completed, in the presence of a researcher, both forms of the ALPHA questionnaire twice, with an interval of one to two weeks. This is a standard time frame in test-retest studies as it is long enough so that respondents are unlikely to remember their answers to the first testing, but short enough to minimise potential changes in physical activity behaviour. To avoid order effects, participants in each study centre were randomly assigned into two groups: Group 1 completed the short version of the questionnaire first (at first and second assessment), followed by the 49-item version, and Group 2 completed the 49-item version first (at first and second assessment), followed by the short version.

To assess predictive validity, physical activity behaviour was measured by accelerometry and long International Physical Activity Questionnaire (IPAQ) last 7 days. Participants were asked to wear accelerometers on the hip during all waking hours for 7 consecutive days following the first visit. Accelerometer recordings were collected at the second visit at which time the researcher interview-administered the Long IPAQ last 7 day. The interview version was preferred to the self-administered version of the IPAQ because of the tendency towards over reporting of physical activity that has been previously reported [18]. The length of time needed to complete each questionnaire at the first visit was recorded. No incentive was provided for participation.

#### Measures

The development of the initial ALPHA environmental questionnaire has been described elsewhere [14]. The instrument included questions on: types of residences in your neighbourhood (3 items), distance to local facilities (8 items), walking or cycle infrastructure in your neighbourhood (4 items), maintenance of infrastructure in your neighbourhood (3 items), neighbourhood safety (6 items), how pleasant is your neighbourhood (4 items), cycling and walking network (4 items), home environment (6 items), workplace or study environment (11 items). For the short form of the questionnaire the number of items was reduced to eleven, with a minimum of one item included from each theme. In both versions neighbourhood was defined as "...the area ALL around your home that you could walk to in 10-15 minutes - approx 1.5 km" (or "1 mile" for UK-context).

Self-reported physical activity level was assessed by the Long IPAQ last 7 day http://www.ipaq.ki.se/ipaq.htm. This instrument asks about physical activity behaviour over the last 7 days, according to categories of physical activity intensity, in different contexts such as physical activity as transport, physical activity at work or study, physical activity at home and physical activity in leisure time; it has been shown to be reliable and valid [19].

The MTI Actigraph accelerometer model 7164 was used in Belgium and France, and the Actigraph GT1M was used in the UK. In all cases an epoch time of one minute was used to provide an objective measure of habitual physical activity (over 7 days).

Finally, participants were asked to provide information on their age, height, weight, sex, ethnicity, living situation, educational attainment, occupational status and living environment.

#### Data reduction

Adverse items of the environmental questionnaire were recoded and sum scores for each scale were calculated.

For the long IPAQ last 7 day, each activity was expressed in minutes/week by multiplying frequency (day/week) and duration (minutes/day) of the activity. Indices of each domain were calculated by summing all physical activities undertaken for each specific context (work, domestic, transport and leisure). A 'total moderate-intensity and vigorous-intensity physical activity' index was computed by summing all reported physical activities undertaken at moderate and vigorous intensity across the four domains.

Accelerometer data were downloaded by placing the accelerometer into a reader interface unit (RIU) and using specific software (RIU256.exe) [20]. Further the data were analysed by a custom-written program (*MAHUFFE*.exe, available from http://www.mrc-epid.cam.ac.uk). Accelerometer data were included in the analysis if the minimal number of wearing days was 4 (with at least one weekend day), with a minimum of 10 hours recording time for week days and 8 hours for weekend days, and excluding the relevant hours if there was an interruption in wearing time during the day of more than 60 minutes. To calculate physical activity at low intensity (LPA), at moderate (MPA) and at vigorous physical activity (VPA) Freedson's cut-offs [21] were used (<1952 counts per minute for LPA, between 1952 -5724 counts per minute for MPA and >5724 counts per minute for VPA).

#### Statistical analysis

Cronbach alphas were calculated to assess the internal consistency of each scale of the environmental questionnaire; results >0.70 were considered good [22]. Intraclass coefficients (sum scores or items on 5 point scales) were used to compute the coefficient of stability of the scores on the two tests. ICC estimates >0.75 were considered as good reliability scores, between 0.50-0.75 as moderate reliability and <0.50 as poor reliability [23]. Proportion of agreement was also calculated to measure the proportion of occasions that individuals gave the same score. Proportion of agreement above 0.70 was considered high [24].

Pearson correlations between environmental variables (sum scores) and accelerometer data, and between environmental variables and IPAQ measurements, were calculated to assess predictive validity.

All analyses were performed using SPSS 15.0 software (SPSS Inc., Chicago, IL, USA).

#### International expert meeting

After the first field testing an international expert meeting in February 2009 was organised to discuss the results (a list of all experts can be found in additional file 1). Items with lower scores on reliability or validity were discussed and rephrased until consensus was reached.

### Field testing II

#### Participants and procedures

For the second and smaller field testing a new sample was recruited in three countries (Belgium, UK and Austria) between April and May 2009 using the same inclusion criteria as in the first field testing. In Belgium a random sample in three different neighbourhoods (town, outskirts of town, and village/countryside - all different from those in the first field testing) was recruited using the same approach as used in the first field testing. In the UK and Austria, convenience samples comprised university colleagues, students and other associates participated. The final sample consisted of 166 participants, 60 from Belgium, 57 from the UK and 49 from Austria.

In this second round of testing only test-retest stability was assessed for both versions, in a similar way to the first field testing.

#### Measures

An adapted version of the ALPHA environmental questionnaire was used. This instrument can be found in additional file 2 (49-item version) and additional file 3 (short version) and on the International Physical activity and Environmental Network (IPEN) website http://www.ipenproject.org. The same themes as in the original version [14] were used, but some items were changed. For example the answer categories of the short version changed from a four point scale (strongly disagree to strongly agree) to a two point scale (yes-no). The neighbourhood definition was also rephrased, reducing the area around the home to "approximately one kilometer or half a mile" instead of 1.5 kilometer and 1 mile. All changes are detailed in additional file 4. No other measures were included in the second field testing.

#### Data reduction and statistical analysis

Adverse items of the environmental questionnaire were recoded and sum scores for each scale were made. Cronbach alphas were calculated to assess the internal consistency of each scale of the environmental questionnaire. Intraclass correlation coefficients (sum scores or items on 5 point scales) and proportion of agreement (separate items) were used to compute the coefficient of stability of the scores on the two tests.

## Results

### Field testing I

Most participants in the first field testing were female (63%); most participants lived in an urban area (86.3%) and were employed (78.9%). Average age was 40 years and average BMI 25 kg/m^2 ^(see Table 1). Cronbach alphas ranged from 0.57-0.76 (data not shown) except for the walking and cycling infrastructure scale (alpha = 0.37).

**Table 1:** Characteristics of the participants in the first and second field testing

	First field testing N = 190		Second field testing N = 166	
**Age (years)**	40.52 ± 12.46		32.89 ± 12.89	
**BMI (kg/m**^**2**^)	25.19 ± 4.37		23.94 ± 3.56	
	**N**	**%**	**N**	**%**
**Sex**				
Male	71	37.4	88	53
Female	119	62.6	78	47
				
**Nationality**				
Belgian	60	31.6	58	35.2
British	66	34.7	56	33.9
French	64	33.7	-	-
Austrian	-	-	49	29.7
**Living situation**				
with partner and children	69	36.3	53	31.9
with partner	46	24.2	37	22.3
with children	20	10.5	2	1.2
with parents	30	15.8	34	20.5
Alone	25	13.2	26	15.7
with friends/other		-	14	8.4
**Higher education**				
Yes	95	50	135	81.3
No	95	50	31	18.7
**Having a job**				
Yes	150	78.9	98	59.4
No	40	21.1	67	40.6
**NS-SEC**				
Managerial and professional occupations	40	24.5	23	23.5
Intermediate occupations	84	51.5	65	66.3
Routine and manual occupations	39	23.9	10	10.2
**Place of residence**				
Town	103	54.2	77	46.4
Outskirts of the town	61	32.1	51	30.7
Village/Countryside	26	13.7	38	22.9

NS-SEC: National Statistics Socio-economic Classification

#### Feasibility

Mean (±SD) time for questionnaire completion during the first assessment was 6 minutes 47 seconds (±2 min) for the 49-item version and 1 minute and 46 seconds (±39 seconds) for the short version.

#### Test-retest reliability

Table 2 shows answer frequencies and mean score of each item on the first assessment of the ALPHA environmental questionnaire and its test-retest reliability scores. The ICCs of the sum scores of each of the nine subscales ranged from 0.66 to 0.86. Six of the nine sum scores were above 0.75 which indicates good reliability; three of them (residential density, infrastructure and maintenance) were between 0.60-0.75, which shows moderate reliability. ICC of the individual items ranged from 0.44-0.82 with the lowest scores for particular safety items and items of the cycling and walking network scale. Proportion of agreement for all individual items ranged from 52-99%.

**Table 2:** First field testing of the ALPHA questionnaire (N = 190): Answer frequencies, mean scores, and test-retest reliability scores.

	Answer Frequencies and mean score of each item on the first assessment						Test-retest reliability scores	
	**Types of residences**							
			
**Item/scale**	**None**	**A Few**	**Some**	**Most**	**All**	**Mean (SD)**	**Agreement, %**	**ICC**

1. Residential density								0.68^a^
a) Detached houses	19.5	31.1	22.6	18.4	8.4	2.7 (1.2)	70	0.80
b) Semi-detached townhouses, terraced houses	8.5	29.6	19.6	37.6	4.8	3.0 (1.1)	66	0.71
c) Flats of 6 floors or more	43.0	24.7	15.1	12.9	4.3	2.1 (1.2)	66	0.72

	Travel time to facilities							
		
	1-5 min	6-10 min	11-20 min	21-30 min	>30 min	Mean (SD)	Agreement, %	ICC

2. Distance to local facilities								0.86 ^b^
a) Local shop	64.7	25.8	7.9	0.5	1.1	1.5 (0.8)	76	0.74
b) Supermarket	20.5	24.7	26.3	9.5	8.9	2.5 (1.2)	67	0.78
c) Local services	20.6	31.2	30.2	11.1	6.9	2.5 (1.1)	66	0.80
d) Restaurant	46.8	27.9	16.8	3.2	5.3	1.9 (1.1)	62	0.71
e) Fast-food restaurant	27.4	25.3	16.3	14.2	16.8	2.7 (1.4)	65	0.82
f) Busstop	74.2	17.4	6.8	0.5	1.1	1.4 (0.7)	81	0.75
g) Sport and leisure facility	16.3	25.3	28.9	14.2	15.3	2.9 (1.3)	63	0.70
h) Open recreation area	37.4	24.2	23.2	8.4	6.8	2.2 (1.2)	59	0.73

	Acceptance of statements							
		
	Strongly disagree	Somewhat disagree	Somewhat agree	Strongly agree	I don't know	Mean (SD)	Agreement, %	ICC

3. Total Infrastructure								0.68 ^b^
Cycling Infrastructure,								0.67 ^c^
Walking infrastructure								0.66 ^d^
a) special lanes, routes or paths to cycle	18.4	13.7	37.9	28.9	1.1	2.8 (1.1)^#^	64	0.75
b) traffic-free cycle routes	41.6	24.2	19.5	12.6	2.1	2.0 (1.1) ^#^	59	0.60
c) sidewalks	2.6	5.8	18.4	72.6	0.5	3.6 (0.7) ^#^	71	0.60
d) pedestrian zones for shopping	55.3	11.6	16.8	14.7	1.6	1.9 (1.2) ^#^	64	0.69

4. Maintenance								0.66^b^
a) cycling paths are well maintained	9.5	10.1	39.7	20.6	20.7	2.9 (0.9) ^#^	60	0.61
b) sidewalks are well maintained	5.3	18.5	56.1	19.0	1.1	2.9 (0.8) ^#^	65	0.58
c) public recreation facilities are well maintained	5.3	9.5	47.1	32.8	5.3	3.1 (0.8) ^#^	62	0.61

	Strongly disagree	Somewhat disagree	Somewhat agree	Strongly agree		Mean (SD)	Agreement, %	ICC

5. Safety								0.75^b^
Safety from crime								0.77^e^
Safety from traffic								0.69^f^
a) not safe to leave a bicycle locked	16.5	28.7	33.5	21.3		2.6 (1.0)	53	0.53
b) not enough safe places to cross busy streets	18.0	46.0	31.2	4.8		2.2 (0.8)	52	0.44
c) Walking is unsafe because of the traffic	35.4	50.3	12.7	1.6		1.8 (0.9)	64	0.54
d) Cycling is unsafe because of the traffic	20.6	44.4	27.5	7.4		2.2 (0.9)	61	0.64
e) unsafe during the day because of the level of crime	58.2	31.7	9.0	1.1		1.5 (0.7)	71	0.61
f) unsafe during the night because of the level of crime	37.0	32.8	22.2	7.9		2.0 (1.0)	68	0.81

6. Pleasant								0.81^b^
Aesthetics								0.79^g^
a) a pleasant environment for walking and cycling	2.1	21.6	52.6	23.7		2.9 (0.7)	69	0.70
b) generally free from litter or graffiti	10.5	29.5	43.7	16.3		2.7 (0.9)	66	0.61
c) trees along the streets	12.6	16.8	33.7	36.8		2.9 (1.0)	62	0.73
d) a lot of badly maintained, unoccupied or ugly buildings	38.4	35.8	20.5	5.3		1.9 (0.9)	66	0.65

7. Cycling & walking network								0.80^b^
Connectivity								0.92^h^
a) Cycling is quicker than driving during the day	20.0	28.4	27.9	15.3	8.4	2.4 (1.0) ^#^	53	0.57
b) Easier to take shortcuts with a bicycle or walking that with a car	10.5	16.3	41.6	27.9	3.7	2.9(0.9) ^#^	57	0.52
c) many intersections	5.3	21.6	43.7	26.3	3.2	2.9 (0.8) ^#^	60	0.61
d) many different routes for cycling or walking from place to place	2.1	23.2	48.9	22.6	3.2	3.0 (0.7) ^#^	66	0.65

	Availability of						Agreement, %	ICC
		
	yes	no						

8. Home environment								0.86^b^
a) Bicycle	61.1	38.9					99	
b) Garden	72.0	28.0					96	
c) Small sports equipment	70.0	30.0					94	
d) Eercise equipment	47.9	52.1					93	
e) Car	82.1	17.9					98	
f) Dog	24.7	75.3					99	

9. Workplace or study environment								0.82^b^
a) Escalators	61.0	39.0					91	
b) Stairs	89.9	10.1					94	
c) Fitness centre/equipment	27.7	72.3					93	
d) Bicycles provided by employer or school	13.8	86.2					93	
e) A safe place to leave a bike	66.5	33.5					85	
f) A free car park	65.4	34.6					90	
g) Showers and changing rooms	57.9	42.1					92	
h) Exercise classes	22.6	77.4					92	
i) Sports club	22.0	48.0					92	
j) Employer subsidised public transport/cycling	44.7	55.3					85	

# without 'I don't know' variable

^a ^weighted by the following formula = item1a + (12* item 1b)+ (50*item 1c); ^b^: total sum score of all items; ^c^: sum score of items 3a & 3b; ^d^: sum score of items 3c & 3d; ^e^: sum score of reversed items 5a, 5e & 5f; ^f^: sum score of reversed items 5b, 5c, 5d; ^g^:sum score of items 6b, 6c, reversed 6d; ^h^:sum score of items 7b, 7c & 7d

Table 3 summarises the answer frequencies and mean scores for each item on the first assessment of the ALPHA short, together with test-retest reliability scores (ICC and proportion of agreement). The ICC of the total sum score was 0.75 which indicates good test-retest stability. The ICC for individual items ranged from 0.50-0.80 and thus showed only moderate reliability. Proportion of agreement was also low ranging from 50-83%, with only two items equal or above 70%.

**Table 3:** First field testing of the ALPHA short (N = 190): Answer frequencies, mean scores, and test-retest reliability scores.

	Answer Frequencies and mean score of each item on the first assessment						Test-retest reliability scores	
**Item/scale**	**Strongly disagree**	**Somewhat disagree**	**Somewhat agree**	**Strongly agree**	**I don't know**	**Mean (SD)**	**Agreement, %**	**ICC**

Total								0.75^a^
A. Most of the houses in my neighbourhood are detached houses	35.8	22.6	24.2	17.4		2.2 (1.1)	66	0.80
B. Many shops, stores, markets or other places to buy things I need are within easy walking distance of my home	4.2	14.2	33.2	48.4		3.9 (0.9)	69	0.64
C. There is a transit stop (such as bus stop, train, trolley or tram station) within easy walking distance of my home	1.6	3.7	15.8	78.9		3.7 (0.6)	83	0.57
D. There is an open recreation area (e.g. park, beach or other open space) within easy walking distance of my home	3.2	11.6	25.3	60.0		3.4 (0.8)	70	0.64
E. There are many different routes for cycling or walking from place to place in my neighbourhood so I don't have to go the same way every time	3.7	18.5	41.3	36.5		3.1 (0.8)	64	0.63
F. Walking and cycling are unsafe because of the traffic in my neighbourhood	19.5	44.2	30.5	5.8		2.2 (0.8)	56	0.51
G. Walking and cycling are unsafe because of the level of crime in my neighbourhood	43.2	40.5	12.1	4.2		1.8 (0.8)	66	0.50
H. My local neighbourhood is a pleasant environment for walking and cycling	3.7	26.3	46.8	26.8		3.0 (0.8)	64	0.57
I. I have access to exercise and sports equipment at home e.g. weights, racquets, skis for personal use	32.1	15.8	22.6	29.5		2.5 (1.2)	61	0.71
J. My workplace provides facilities to support me walking or cycling to work e.g. changing rooms, bike storage	25.3	14.7	23.7	22.1	14.2	2.5 (1.2)^#^	50	0.58
K. I have access to exercise and sports facilities at work e.g. fitness centre/equipment, stairs	38.1	15.9	16.9	15.3	13.8	2.1 (1.1) ^#^	59	0.69

^#^without "not applicable"; ^a^: total sum score of all items

#### Predictive validity

Tables 4 and 5 show the significant correlations of the subscale of the ALPHA questionnaires (both forms) with the physical activity measurements (both IPAQ and accelerometers).

**Table 4:** First field testing (N = 190): Pearson correlations between the ALPHA questionnaire and ALPHA short with long last 7 day IPAQ, stratified by sex (only statistically significant values are shown).

ALPHA & IPAQ									
**Name of the scale (number of items in the questionnaire)**	**Sex**^**a**^	**Total PA**	**MVPA**	**Walking for transport**	**Cycling for transport**	**Total transport**	**Total walking**	**Leisure time PA**	**Home PA**

Density score (1a, 1b, 1c)	Male								
	Female			0.20*			0.21*		

Distance to local facilities (2a to 2h)	Male	-		-0.33**				-0.32**	
	Female	0.25*		-0.20*					

Availability of sidewalks (3c, 3d)	Male			0.32*					
	Female		-0.20*		-0.19*				

Maintenance (4a, 4b, 4c, 4d)	Male					0.30*			
	Female								

Safety from traffic (reversed 5b, 5c, 5d)	Male								
	Female			-0.31**		-0.30**	-0.22*	0.22*	

Aesthetics (6b, 6c, 6d)	Male								
	Female		0.19*	-0.25**		-0.23*			

Cycling and walking network (7a, 7d)	Male						0.24*		
	Female								

Connectivity (7b, 7c, 7d)	Male						0.28*		
	Female								

Home environment (8a to 8f)	Male							0.38**	
	Female								0.20*

Workplace or study environment (9a to 9j)	Male		0.33**		0.25*			0.34**	
	Female								

**ALPHA short & IPAQ**									

**Total sum of 11 items**	Male		0.34**					0.25*	
	Female				0.21*				

^a ^men: N = 70, women: N = 120

Total_PA: total minutes PA/week (low, moderate, vigorous in all domains); MVPA:total minutes PA/week at moderate to vigorous intensity; walking for transport: total minutes transport-related walking/week; Cycling for transport: total minutes transport-related cycling/week; Total transport: total minutes PA/week by active transportation (walking and/or cycling for transport); Total walking: total minutes walking/week (job, transport, leisure time); Leisure time PA:total minutes PA/week in leisure time; Home PA: total minutes PA/week in and around the house (inclusive gardening); * p < 0.05; ** p < 0.01

**Table 5:** First field testing (N = 190): Pearson correlations between ALPHA and ALPHA short questionnaire with accelerometer, stratified by sex (only statistically significant values are shown).

Name of the scale (number of items in the questionnaire)	**Sex**^**a**^	Total PA	VPA	MVPA	LPA
Density score (1a, 1b, 1c)	Male				
	Female				

Distance to local facilities (2a, 2b, 2c, 2d, 2e, 2f, 2g, 2h)	Male				
	Female				

Availability of sidewalks (3c, 3d)	Male				
	Female				

Maintenance (4a, 4b, 4c, 4d)	Male				
	Female				

Safety from traffic (reversed 5b, 5c, 5d)	Male				
	Female		0.20*		

Aesthetics (6b, 6c, 6d)	Male				
	Female	0.25*			0.26*

Cycling and walking network (7a, 7d)	Male				
	Female	0.28**			0.29

Connectivity (7b, 7c, 7d)	Male				
	Female			0.24*	

Home environment (8a, 8b, 8c, 8d, 8e, 8f)	Male				
	Female	0.21*			0.21*

Workplace or study environment (9a, 9b, 9c, 9d, 9e, 9f, 9h, 9i, 9j)	Male				0.32*
	Female	0.34**			0.32**

**ALPHA short & accelerometer**					

Total sum of 11 items	Male				0.29*
	Female	0.26**		0.28**	0.28*

^a ^men: N = 65: women: N = 106

Total_PA: total minutes PA/week (low, moderate, vigorous in all domains); VPA: total minutes PA/week at vigorous intensity (>5724 counts per minute); MVPA:total minutes PA/week at moderate to vigorous intensity (>1951 counts per minute); LPA: total minutes PA/week at low intensity (below 1952 counts per minute); * p < 0.05; ** p < 0.01

All significant correlations were in the hypothesised directions (higher environmental support of physically activity was correlated with higher levels of physically activity) except for the negative correlations found between the scales 'availability of sidewalks' and 'safety from traffic' with some IPAQ variables. The size of all correlations ranged from 0.19-0.38 which is an indication of moderate validity. Environmental scales of ALPHA were mostly significantly correlated with minutes of transport-related walking as measured with the IPAQ, both in men and women. Very few significant correlations were found with accelerometers in men, however there were several significant correlations found in women, especially with physical activity at low intensity.

The sum score calculated from the ALPHA short was significantly correlated with both IPAQ and accelerometers in men and women. All significant correlations were in the expected directions and ranged from 0.21-0.34.

#### International expert meeting

Based on the results of the first field testing wording and answer categories of specific items with lower reliability scores were modified following discussions at the expert meeting (see Additional file 4).

### Field testing II

In the second field testing almost half (47%) of the participants were female. Most of the participants lived in an urban area (77.1%), and were employed (59%), with an average age of 33 years and an average BMI of 24 kg/m2 (see Table 1).

#### Internal consistency

Cronbach alphas ranged from 0.65-0.82 except for the pleasant environment scale (alpha = 0.34) (data not shown).

#### Test-retest reliability

Answer frequencies and mean scores for each item on the first assessment of the ALPHA questionnaire and their test-retest reliability scores are shown in Table 6. ICCs of the sum scores of each subscale ranged from 0.71 to 0.87, with six of the nine ICCs above 0.75, showing good test-retest reliability. ICCs of the individual items ranged from 0.54-0.87, showing moderate to good stability. Proportions of agreement for all individual items ranged from 59-99%.

**Table 6:** Second field testing of the ALPHA questionnaire (N = 166): Answer frequencies; mean scores and its test-retest reliability scores.

	Answer Frequencies and mean score of each item on the first assessment						Test-retest reliability scores	
**Item/scale**	**None**	**A Few**	**Some**	**Most**	**All**	**Mean (SD)**	**Agreement %**	**ICC**

1. Residential density								0.80^a^
a) Detached houses	18.5	27.8	27.8	22.2	3.7	2.65 (1.1)	77	0.81
b) Semi-detached houses or terraced houses	3.7	15.2	39.0	39.0	3.0	3.23 (0.9)	59	0.56
c) Apartment buildings or blocks of flats	22.0	37.8	27.4	11.6	1.2	2.32 (1.0)	73	0.80

	Travel time to facilities							

	1-5 min	6-10 min	11-20 min	21-30 min	>30 min	Mean (SD)	Agreement %	ICC

2. Distance to local facilities								0.87^b^
a) Local shop	57.2	22.9	13.9	3.0	3.0	1.7 (1.0)	75	0.80
b) Supermarket	43.0	24.2	20.6	5.5	6.7	2.1 (1.2)	75	0.82
c) Local services	30.7	29.5	21.7	12.0	6.0	2.3 (1.2)	67	0.77
d) Restaurant, café, pub..	50.0	30.1	12.0	4.8	3.0	1.8 (1.0)	75	0.72
e) Fast-food restaurant	36.2	23.3	13.8	12.1	14.7	2.5 (1.5)	60	0.87
f) Bus stop	72.7	17.6	5.5	3.0	1.2	1.4 (0.8)	79	0.74
g) Sport and leisure facility	18.7	29.5	27.7	11.4	12.7	2.7 (1.2)	65	0.75
h) Open recreation area	48.5	27.9	10.3	6.1	7.3	3.5 (0.8)	76	0.76

	Acceptance of statements							

	Strongly disagree	Somewhat disagree	Somewhat agree	Strongly agree		Mean (SD)	Agreement %	ICC

3. Infrastructure								0.79^b^
Pedestrian infrastructure								0.75^c^
Cycling infrastructure								0.74^d^
a) Sidewalks	4.8	7.8	17.5	69.9		3.5 (0.8)	73	0.74
b) pedestrian zones or pedestrian trails	24.7	23.5	28.9	22.9		2.5 (1.1)	59	0.65
c) Special lanes, routes or paths for cycling	18.8	17.0	33.9	30.3		2.8 (1.1)	62	0.69
d) cycle routes separated from traffic	26.7	24.2	28.5	20.6		2.4 (1.1)	61	0.68

	Strongly disagree	Somewhat disagree	Somewhat agree	Strongly agree	Not applicable	Mean (SD)	Agreement %	ICC

4. Maintenance								0.74^#*b*^
a) sidewalks are well maintained	7.9	13.4	42.1	35.4	1.2	3.1 (0.9	57	0.60^#^
b) cycling paths are well maintained	15.3	13.5	40.5	23.9	6.7	2.8 (1.0)	63	0.55^#^
c) Play areas, playgrounds, parks or other open spaces are well maintained	3.1	11.7	47.2	35.0	3.1	3.2 (0.8)	66	0.62^#^

	Strongly disagree	Somewhat disagree	Somewhat agree	Strongly agree		Mean (SD)	Agreement %	ICC

5. Safety							0.71 ^b^	
Safety from crime							0.78 ^e^	
Safety from traffic							0.68 ^f^	
a) dangerous to leave a bicycle locked	28.5	40.0	20.6	10.9		2.1 (1.0)	66	0.69
b) not enough safe places to cross busy streets	41.2	37.0	18.2	3.6		1.8 (0.8)	61	0.57
c) Walking is dangerous because of the traffic	47.3	40.6	10.3	1.8		1.7 (0.7)	68	0.58
d) Cycling is dangerous because of the traffic	35.8	37.0	24.2	3.0		2.0 (0.9)	62	0.61
e) dangerous during the day because of the level of crime	73.9	23.0	2.4	0.6		1.3 (0.5)	74	0.57
f) dangerous during the night because of the level of crime	43.1	44.0	8.6	4.3		1.7 (0.8)	69	0.60

6. Pleasant								0.84 ^b^
Aesthetics								0.74 ^g^
a) a pleasant environment for walking and cycling	3.6	19.3	45.2	31.9	3.1 (0.8)		68	0.65
	
	None	A few	Some	Plenty		Mean (SD)	Agreement %	ICC
	
b) litter or graffiti	36.7	34.9	20.5	7.8		2.0 (0.9)	71	0.82
c) trees along the streets	15.1	18.7	37.3	28.9		2.8 (1.0)	66	0.72
d) badly maintained, unoccupied or ugly buildings	40.6	44.2	9.7	5.5		1.8 (0.8)	68	0.57

	Strongly disagree	Somewhat disagree	Somewhat agree	Strongly agree		Mean (SD)	Agreement %	ICC

7. Cycling and walking network								0.84 ^b^
Connectivity								0.91^h^
a) Many shortcuts for walking	6.0	27.7	47.0	19.3		2.8 (0.8)	67	0.58
b) Cycling is quicker than driving during the day	19.9	31.9	25.9	22.3		2.5 (1.0)	67	0.79
c) many road junctions	7.2	35.5	39.8	17.5		2.7 (0.8)	61	0.61
d) many different routes for cycling or walking from place to place	3.6	19.3	56.0	21.9		2.9 (0.7)	63	0.54

	Availability of							

	yes	no					Agreement %	ICC

8. Home environment								0.72 ^b^
a) Bicycle	81.3	18.7					99	
b) Garden	76.5	23.5					95	
c) Small sports equipment	86.7	13.3					94	
d) Exercise equipment	60.2	39.2					96	
e) Car	86.1	13.3					98	
f) Dog	19.3	80.1					98	

9. Workplace or study environment								0.83 ^b^
a) Escalators	47.4	52.6						91
b) Stairs	89.5	10.5						95
c) Fitness centre/equipment	34.9	65.1						91
d) Bicycles provided by employer or school	19.9	80.1						91
e) A safe place to leave a bike	74.2	25.8						87
f) Enough car parking spaces	56.3	43.7						93
g) Showers and changing rooms	62.9	37.1						93
h) Exercise classes	39.1	60.9						91
i) Sports club	38.4	61.6						89
j) Employer subsidised public transport	48.3	51.7						87

# without not applicable

ICC = Intraclass correlation coefficient

^a ^weighted by the following formula = item1a + (12* item 1b)+ (50*item 1c); ^b^: total sum score of all items; ^c^: sum score of items 3a & 3b; ^d^: sum score of items 3c & 3d; ^e^: sum score of reversed items 5a, 5e & 5f; ^f^: sum score of reversed items 5b, 5c, 5d; ^g^:sum score of items 6b, 6c, reversed 6d; ^h^:sum score of items 7a, 7c & 7d

In Table 7 the answer frequencies of each item on the first assessment of the ALPHA short were given, together with their test-retest reliability scores (ICC for the sum scores and proportions of agreement for the individual items). The ICC of the total sum score of the ALPHA short was 0.73 which indicates good test-retest stability. For the individual items the proportions of agreement were good, ranging from 85 to 95%.

**Table 7:** Second field testing of the ALPHA short (N = 166): Answer frequencies and test-retest reliability scores.

	Answer frequencies			Test-retest reliability scores	
Item/Scale	Yes	No	NA	Agreement, %	ICC
Total					0.73^a^
a) Most of the houses in my neighbourhood are detached houses	33.7	66.3		89	
b) There are many shops within easy walking distance of my home	74.7	25.3		92	
c) There is a bus/tram station within easy walking distance of my home	92.2	7.8		92	
d) There is a park within easy walking distance of my home	84.9	15.1		92	
e) Walking is dangerous because of the traffic in my neighbourhood	7.8	92.2		90	
f) Walking is dangerous because of the level of crime in my neighbourhood	4.2	95.8		95	
a) There are trees along the streets in my neighbourhood	67.6	32.3		85	
g) At my home, I have small sports equipment such as a ball, racquets, ... for my personal use	82.5	17.5		92	
h) At my work or place of study I have bicycles provided by employer or school	10.8	77.1	12.0	93	
i) At my work or place of study I have employer subsidized public transport	41.0	47.0	12.0	90	

NA = Not applicable

ICC = Intraclass correlation coefficient

^a^sum score of all items

## Discussion

The purpose of this study was to assess test-retest reliability, predictive validity and feasibility of the ALPHA environmental questionnaire in samples of men and women from several European countries.

### Reliability

All but two of the subscales (distance to local facilities scale and the safety scale) in the ALPHA questionnaire showed low levels of internal consistency. This is appropriate for environmental variables as the aim of an environmental questionnaire is to sample possible indicators of one environmental construct which are often not intercorrelated, so Cronbach alphas are often low. In the literature similar internal consistency values for environmental scales are found e.g. the Cronbach alphas of the Cycling for Transport questionnaire range from 0.46 to 0.70 [25].

In the first testing, moderate to good test-retest reliability was evident for the ALPHA questionnaire (ICCs of the subscales ranged from 0.66-0.86); while in the second field testing all subscales showed good reliability (ICCs ranged from 0.71-0.87). The ICCs were not significantly different between two test-phases (t = -1.207, p = 0.247), but there was a significant increase (t = -2.779, p = 0.008) in percentages of agreement from the first (range 52-99%) to the second field testing (range 59-99%). Similar test-retest values have been reported for other environmental questionnaires, e.g. ICCs for the test-retest reliability of the NEWS subscales ranged from 0.58 to 0.80 in one study [12] and from 0.41-0.93 in another study [26]; for the IPAQ environmental module ICCs ranged from 0.36 to 0.98 [27]; and Evenson et al. reported ICC values from 0.64 to 0.91 for environmental items in their physical activity questionnaire [28].

Overall, our findings suggest that the final version of the ALPHA questionnaire has good reliability, comparable to that found in equivalent instruments.

The reliability results of the ALPHA short were more difficult to compare between both field tests, given the changes to answer categories (i.e. ICCs for the individual items could not be analysed). However, for the total score, reliability was good and of similar magnitude in both testing phases (0.75 and 0.73 respectively). For the individual items the proportion of agreement found in the first field testing (50% to 83%) increased significantly (t = -9.175, p < 0.001) in the second field testing (85% to 95%); showing greater item stability in the later version. Reliability values of the ALPHA short compared favourably with other instruments, e.g. proportion of agreement for environmental items of South Carolina (47% to 94%), for the NEWS (33% to 98%) and for the St Louis Instrument (40% to 96%) [26].

### Predictive validity

In general, moderate predictive validity was found for both versions of the ALPHA environmental questionnaire. As expected, most associations were found between the environmental scales and "walking for transport" measured with the IPAQ. This is consistent with the transport literature in which urban planners show that certain environmental factors, like those included in the ALPHA questionnaire, are associated with increased levels of walking [29,30]. Also Saelens et al. [12] and De Bourdeaudhuij et al. [31] have found good associations between the attributes of built environment and walking for transport. It should be mentioned, however, that because of the cross-sectional nature of the current and previous studies [8,32], no causal conclusions could be drawn. Therefore, the explanation that the built environment has a positive influence on physical activity levels could also be reversed i.e. people with higher physical activity levels perceive more physical activity opportunities in their built environment than people who are less physically active.

With the accelerometers, context-related physical activity could not be assessed, but almost all associations between the perceived environment and low physical activity were in line with the results of the IPAQ. Contrasting results between IPAQ and accelerometers were found for "safety from traffic" and "aesthetics". Somewhat unexpectedly, these environmental factors were related with lower levels of walking for transport measured with the IPAQ in women. However, we did find associations in the hypothesised direction with the Actigraph data, namely "safety from traffic" and "aesthetics" were related with respectively more minutes of vigorous physical activity and more minutes of physical activity at low intensity. In the literature, aesthetics and physical activity behaviour are consistently positively related, but the association between safety and physical activity behaviour is less consistent [8].

### Feasibility

Both versions of the questionnaire appeared feasible in terms of completion time. The 49-item version was completed in a relatively short period of time compared to other environmental questionnaires requiring only an average of 6 minutes to complete. Given the low participant burden we recommend using this version as it gives a better overall picture of the built environment than ALPHA short.

### Strengths and Limitations

The ALPHA questionnaire has undergone extensive conceptual and field testing and refinement. It is now ready for further assessment within different populations and environments across Europe.

One of the limitations in this study was the high education level of our participants in the second series of field testing compared with our first field test participants, which might explain some of the improvements seen in the test-retest scores.

A second limitation of this study are the different sampling methods (probability and non-probability) used in the three countries and the possible clustering within each country, which may have resulted in more positive results.

Another possible limitation is that objective environmental measurements were not included in the testing and thus the perceptions could not be compared with objective data. However, it has to take into account that objective and subjective measures of the build environment are two different concepts. Previous studies [33-35] found only a low to moderate agreement between objective and subjective measures. In some studies perceptions of the environment had a greater impact on PA behavior (or vice versa) compared to objective measured environment [33,36] while another study found a greater influence of objective measures [37]. More research is needed to explore further the relationships and differences between perceived and objectively measured attributes of the environment.

Our questionnaire was based on extensive synthesis and adaptation of previous similar instruments [14] however this may repeat any systematic errors contained within these instruments [14,38]. We feel there remains a challenge in built environment and physical activity research of evaluating the congruence between definitions used in environmental questionnaires and adults' own definitions of neighbourhood.

## Conclusion

The ALPHA questionnaire is a good instrument for measuring environmental perceptions related to physical activity behaviour, with moderate to good reliability, predictive validity and feasibility. The instrument was developed in collaboration with an international expert group and was subject to different test phases. However, we acknowledge the considerable challenges of this field, and in light of the limitations outlined believe that further testing is required to improve generalisability to other European countries. Future testing will look to correlate the perceived environmental outcomes with other physical activity-related measures such as fitness, heart rate and geographic information system (GIS) measured objective environmental measures. By the means of this paper we would also like to make the ALPHA questionnaire available to other researchers who could further investigate whether our questionnaire represents an appropriate instrument for assessing perceptions of the environment related to physical activity across Europe. The questionnaire (in different languages) and a manual can be found on the IPEN website http://www.ipenproject.org.

## Competing interests

The authors declare that they have no competing interests.

## Authors' contributions

MS, PO, IDB, HR, CF, JMO and HS identified the research question and design of this study as part of the ALPHA project. MV, JG, CG and ST collected the data. HS coordinated translation process and data collection, did the statistical analysis and drafted the manuscript. All authors contributed to synthesising the results and critical revision of the manuscript, and approved the final version.

## Supplementary Material

Additional file 1 **International expert group**. List of the members of the international expert group. Click here for file

Additional file 2 **ALPHA questionnaire**. ALPHA measure of environmental perceptions: active travel and physical activity. For different languages and a manual see also http://www.ipenproject.org. Click here for file

Additional file 3 **ALPHA questionnaire (short form)**. ALPHA short measure of environmental perceptions: active travel and physical activity. Click here for file

Additional file 4 **Adaptations**. Adaptations of ALPHA and ALPHA short, made after the first field testing and second expert meeting Click here for file
