# Supplementary material for: Measuring physical activity-related environmental factors: reliability and predictive validity of the European environmental questionnaire ALPHA
Source: Int J Behav Nutr Phys Act. 2010 May 26;7:48. doi: 10.1186/1479-5868-7-48 (PMC2892430; doi:10.1186/1479-5868-7-48)
Supplement: Additional file 1 — International expert group. List of the members of the international expert group. [file 1479-5868-7-48-S1.PDF]

International expert group

- Klaus Gebel (Germany)
- David Ogilvie (UK)
- Frank Van Lenthe (Netherlands)
- Patrick Bergman (Sweden)
- Kristina Sundquist (Sweden)
- Roger Macket (UK)
- Janet Klara Djomba (Slovenia)
- Maria Hagstromer (Sweden)
- Andy Jones (UK)
- Rachel Davy (UK)
