# Supplementary material for: Measuring physical activity-related environmental factors: reliability and predictive validity of the European environmental questionnaire ALPHA
Source: Int J Behav Nutr Phys Act. 2010 May 26;7:48. doi: 10.1186/1479-5868-7-48 (PMC2892430; doi:10.1186/1479-5868-7-48)
Supplement: Additional file 2 — ALPHA questionnaire. ALPHA measure of environmental perceptions: active travel and physical activity. For different languages and a manual see also http://www.ipenproject.org. [file 1479-5868-7-48-S2.PDF]

ALPHA measure of environmental perceptions: active travel and physical activity

We would like to find out more information about the way that you think about your neighbourhood, home environment and workplace or study environment. Please answer as honestly and completely as possible and provide only one answer for each item. There are no right or wrong answers and your information will be kept confidential.

1. Types of residences in your neighbourhood

How common are the following types of residences in your immediate neighbourhood?

By your neighbourhood we mean ALL the area within approximately one kilometer or half a mile of your home or that you could walk to in 10-15 minutes.

Please put one check mark (✓) per answer that best applies to your view of your neighbourhood

	None	A few	Some	Most	All
a) Detached houses					
b) Semi-detached houses or terraced houses					
c) Apartment buildings or blocks of flats					

2. Distance to local facilities

About how long would it take to get from your home to the *<u>nearest</u>* businesses or facilities listed below if you WALKED to them?
Please put <u>one</u> check mark (✓) for each business or facility.

The nearest…	1-5 min	6-10 min	11-20 min	21-30 min	More than 30 min
a) Local shop: grocery shop, bakery, butcher etc.					
b) Supermarket					
c) Local services such as a bank, post office or library, …					
d) Restaurant, café, pub or bar					
e) Fast-food restaurant or takeaway					
f) Bus stop, tram, metro or train station					
g) Sport and leisure facility such as a swimming pool, sports field or fitness centre					
h) Open recreation area such as a park or other open space					

3. Walking and cycling infrastructure in your neighbourhood

By your neighbourhood we mean ALL the area within approximately one kilometer or half a mile of your home or that you could walk to in 10-15 minutes.

Please circle one answer per statement

	Strongly disagree	Somewhat disagree	Somewhat agree	Strongly agree
a) There are sidewalks in my neighbourhood	1	2	3	4
b) There are pedestrian zones or pedestrian trails in my neighbourhood	1	2	3	4
c) There are special lanes, routes or paths for cycling in my neighbourhood	1	2	3	4
d) There are cycle routes in my neighbourhood that are separated from traffic	1	2	3	4

4. Maintenance of walking and cycling infrastructure in your neighbourhood

By your neighbourhood we mean ALL the area within approximately one kilometer or half a mile of your home or that you could walk to in 10-15 minutes.

Please circle one answer per statement

	Strongly disagree	Somewhat disagree	Somewhat agree	Strongly agree	Not applicable
a) The sidewalks in my neighbourhood are well maintained	1	2	3	4	5
b) The cycle paths in my neighbourhood are well maintained	1	2	3	4	5
c) The play areas, playgrounds, parks or other open spaces in my neighbourhood are well maintained	1	2	3	4	5

5. Neighbourhood safety

By your neighbourhood we mean ALL the area within approximately one kilometer or half a mile of your home or that you could walk to in 10-15 minutes.

Please circle one answer per statement

	Strongly disagree	Somewhat disagree	Somewhat agree	Strongly agree
a) It is dangerous to leave a bicycle <u>locked</u> in my neighbourhood	1	2	3	4
b) There are not enough safe places <u>to cross</u> busy streets in my neighbourhood	1	2	3	4
c) Walking is dangerous because of the <u>traffic</u> in my neighbourhood	1	2	3	4
d) Cycling is dangerous because of the <u>traffic</u> in my neighbourhood	1	2	3	4
e) It is dangerous in my neighbourhood <u>during the day</u> because of the level of crime	1	2	3	4
f) It is dangerous in my neighbourhood <u>during the night</u> because of the level of crime	1	2	3	4

6. How pleasant is your neighbourhood for walking or cycling?

By your neighbourhood we mean ALL the area within approximately one kilometer or half a mile of your home or that you could walk to in 10-15 minutes.

Please circle one answer per statement

	Strongly disagree	Somewhat disagree	Somewhat agree	Strongly agree
a) My local neighbourhood is a pleasant environment for walking or cycling	1	2	3	4
	None	A few	Some	Plenty
b) There is litter or graffiti in the streets of my neighbourhood	1	2	3	4
c) There are trees along the streets in my neighbourhood	1	2	3	4
d) In my neighbourhood there are badly maintained, unoccupied or ugly buildings	1	2	3	4

7. Walking and cycling network

By your neighbourhood we mean ALL the area within approximately one kilometer or half a mile of your home or that you could walk to in 10-15 minutes.

Please circle one answer per statement

	Strongly disagree	Somewhat disagree	Somewhat agree	Strongly agree
a) There are many shortcuts for walking in my neighbourhood	1	2	3	4
b) Cycling is quicker than driving in my neighbourhood during the day	1	2	3	4
c) There are many road junctions in my neighbourhood	1	2	3	4
d) There are many different routes for walking or cycling from place to place in my neighbourhood so I don’t have to go the same way every time	1	2	3	4

8. Home Environment

	*Please tick **Yes** or **No*** [✓]	
	**Yes**	**No**
a) Do you have a bicycle for your personal use?		
b) Do you have a garden (including a yard, allotment or city garden)?		
c) Do you have small sports equipment such as a ball, racquets, …for your personal use?		
d) Do you have exercise equipment such as weights, treadmill, stationary cycle, …for your personal use?		
e) Do you have access to a car?		
f) Do you have a dog ?		

9. Workplace or study environment

A. How far do you have to travel to get to your usual place of work or study?

I do not work or study → Please skip part B

I usually work at home or from home → Please skip part B

The distance to my work or place of study is _____________miles/kilometres (circle as appropriate)

B. At your work or place of study do you have....?

	Please tick one box only [✓]	
	**Yes**	**No**
a) …escalators or lifts		
b) …stairs		
c) …fitness centre/equipment		
d) …bicycles provided by employer or school		
e) …a safe place to leave a bike		
f) …enough car parking spaces		
g) …showers and changing rooms		
h) …exercise classes (e.g. aerobics classes)		
i) …sports club / association (e.g. running club)		
j) …employer/school subsidised public transport		
