# Supplementary material for: Measuring physical activity-related environmental factors: reliability and predictive validity of the European environmental questionnaire ALPHA
Source: Int J Behav Nutr Phys Act. 2010 May 26;7:48. doi: 10.1186/1479-5868-7-48 (PMC2892430; doi:10.1186/1479-5868-7-48)
Supplement: Additional file 3 — ALPHA questionnaire (short form). ALPHA short measure of environmental perceptions: active travel and physical activity. [file 1479-5868-7-48-S3.PDF]

Assessing Levels of Physical Activity

Short ALPHA measure of environmental perceptions: active travel and physical activity

We would like to find out more information about the way that you think about your neighbourhood, home environment and workplace or study environment.
Please put one check mark (✓) per answer that best applies to your view of your neighbourhood, home environment and workplace or study environment.
By your neighbourhood we mean ALL the area within approximately one kilometer or half a mile of your home or that you could walk to in 10-15 minutes.

	Yes	No	
a) Most of the houses in my neighbourhood are detached houses			
b) There are many shops within easy walking distance of my home			
c) There is a bus/tram station within easy walking distance of my home			
d) There is a park within easy walking distance of my home			
e) Walking is dangerous because of the traffic in my neighbourhood			
f) Walking is dangerous because of the level of crime in my neighbourhood			
g) There are trees along the streets in my neighbourhood			
h) At my home, I have small sports equipment such as a ball, racquets, …for my personal use			
i) At my work or place of study I have bicycles provided by employer or school			NA
j) At my work or place of study I have employer subsidised public transport			NA
