# Supplementary material for: Measuring physical activity-related environmental factors: reliability and predictive validity of the European environmental questionnaire ALPHA
Source: Int J Behav Nutr Phys Act. 2010 May 26;7:48. doi: 10.1186/1479-5868-7-48 (PMC2892430; doi:10.1186/1479-5868-7-48)
Supplement: Additional file 4 — Adaptations. Adaptations of ALPHA and ALPHA short, made after the first field testing and second expert meeting [file 1479-5868-7-48-S4.PDF]

Adaptations of the ALPHA questionnaire and ALPHA short made after the first field testing and expert meeting

ALPHA Question	Original version and changes made	Adapted version
All questions	Rephrased neighbourhood definition:<br>"By your neighbourhood we mean the area ALL around your home that you could walk to in 10-15 minutes - approx 1 mile or 1.5 km"	"By your neighbourhood we mean ALL the area within approximately one kilometer or half a mile of your home or that you could walk to in 10-15 minutes."
1	- rephrased item b:<br>"Semi-detached townhouses, terraced houses"<br>- rephrased item c:<br>"Flats of 6 floors or more"	"Semi-detached houses or terraced houses"<br><br>"Apartment buildings or blocks of flats"
2	rephrased item h<br>"Open recreation area such as a park, beach or other open space"	"Open recreation area such as a park or other open space"
3	- Reprhased title<br>"Walking or cycling infrastructure in your neighbourhood"<br>- Deleted "I don't know" answer category<br>- reordered items<br>- rephrased items b & d<br>a)There are special lanes, routes or paths to cycle in my neighbourhood<br>b)There are traffic-free cycle routes in my neighbourhood<br>c)There are sidewalks in my neighbourhood<br><br>d) There are pedestrian zones in my neighbourhood for shopping	"Walking and cycling infrastructure in your neighbourhood"<br><br><br><br>a) There are sidewalks in my neighbourhood<br><br>b) There are pedestrian zones or pedestrian trails in my neighbourhood<br>c) There are special lanes, routes or paths for cycling in my neighbourhood<br>d) There are cycle routes in my neighbourhood that are separated from traffic
4	- Reprhased title<br>"Maintenance of infrastructure in your neighbourhood"<br>- Reordered items<br>- Changed answer category<br>"I don't know/Not applicable"	"Maintenance of walking and cycling infrastructure in your neighbourhood"<br><br><br><br>"Not applicable"
5	Changed the words "unsafe" and "not safe" into dangerous<br>a) It is not safe to leave a bicycle <u>locked</u> in my neighbourhood<br>c) Walking is unsafe because of the <u>traffic</u> in my neighbourhood<br>d) Cycling is unsafe because of the <u>traffic</u> in my neighbourhood<br>e) It is unsafe in my neighbourhood <u>during the day</u> because of the level of crime<br>f) It is unsafe in my neighbourhood <u>during the night</u> because of the level of crime	a) It is dangerous to leave a bicycle <u>locked</u> in my neighbourhood<br>c) Walking is dangerous because of the <u>traffic</u> in my neighbourhood<br>d) Cycling is dangerous because of the <u>traffic</u> in my neighbourhood<br>e) It is dangerous in my neighbourhood <u>during the day</u> because of the level of crime<br>f) It is dangerous in my neighbourhood <u>during the night</u> because of the level of crime
6	- Rephrased title<br>"How pleasant is your neighbourhood?"<br><br>-Rephrased item a<br>"My local neighbourhood is a pleasant environment for walking and cycling"<br>- Rephrased item b<br>"My neighbourhood is generally free from litter or graffiti"<br>- Items b, c, d other answer categories<br>"Strongly disagree – somewhat disagree –	"How pleasant is your neighbourhood for walking or cycling?"<br><br>"My local neighbourhood is a pleasant environment for walking or cycling"<br><br>"There is litter or graffiti in the streets of my neighbourhood"<br><br>"None – a few – some – plenty"

	somewhat agree – strongly agree"	
7	- Rephrased title<br>"Cycling and walking network"<br>- Item a & b reordered<br>- Item a rephrased<br>"It is easier to take shortcuts with a bicycle or walking than with a car"<br>- Item c rephrased<br>"There are many intersections in my neighbourhood"	"Walking and cycling network"<br><br>"There are many shortcuts for walking in my neighbourhood"<br><br>"There are many road junctions in my neighbourhood"
9	Rephrased Items f & g<br>"A free car park"<br>"employer subsidised public transport / cycling"	"Enough car parking spaces"<br>"employer/school subsidised public transport"

ALPHA short question	Original version and changes made		Adapted version
All items	Answer categories were changed for all items<br>“Strongly disagree –somewhat disagree – somewhat agree – strongly agree”	All items	“Yes – No”
b	Item b rephrased:<br>“Many shops, stores, markets or other places to buy things I need are within easy walking distance of my home”	b	“There are many shops within easy walking distance of my home”
c	Item c rephrased:<br>“There is a transit stop (such as bus stop, train, trolley or tram station) within easy walking distance of my home”	c	“There is a bus/tram station within easy walking distance of my home”
d	Item d rephrased:<br>“There is an open recreation area (e.g. park, beach or other open space) within easy walking distance of my home”	d	“There is a park within easy walking distance of my home”
e	Item e deleted:<br>“There are many different routes for cycling or walking from place to place in my neighbourhood so I don’t have to go the same way every time”		
f	Item f rephrased:<br>“Walking and cycling are unsafe because of the traffic in my neighbourhood”	e	“Walking is dangerous because of the traffic in my neighbourhood”
g	Item g rephrased:<br>“Walking and cycling are unsafe because of the level of crime in my neighbourhood”	f	“Walking is dangerous because of the level of crime in my neighbourhood”
h	Item h was replaced:<br>“My local neighbourhood is a pleasant environment for walking and cycling”	g	“There are trees along the streets in my neighbourhood “
i	Item I rephrased:<br>“I have access to exercise and sports equipment at home e.g. weights, racquets, skis for personal use”	h	“At my home, I have small sports equipment such as a ball, racquets, …for my personal use”
j	Item j was replaced<br>“My workplace provides facilities to support me walking or cycling to work e.g. changing rooms, bike storage”	i	“At my work or place of study I have bicycles provided by employer or school”
k	Item k was replaced<br>I have access to exercise and sports facilities at work e.g. fitness centre/equipment, stairs”	j	“At my work or place of study I have employer subsidised public transport”
